# A thematic analysis of bereaved adults' meaning-making experience of loss through playing video games

**DOI:** 10.3389/fpsyg.2023.1154976

**Published:** 2023-07-31

**Authors:** Karam Eum, Young Yim Doh

**Affiliations:** Games and Life Lab, Graduate School of Culture Technology, Korea Advanced Institute of Science and Technology (KAIST), Daejeon, Republic of Korea

**Keywords:** bereavement, video games, meaning-making, thematic analysis, reflection, autobiographical memory

## Abstract

**Introduction:**

Recalling personal memories on the loss and deriving new meanings from them is deemed necessary for adapting to bereavement. Recent studies suggest that games can afford players meaningful experiences that can support players through stressful life events, but its potential on the meaning-making of loss has not been much explored. To address this gap, we investigated the bereaved players' experiences of playing commercial video games that elicited their personal memories of loss and what meanings they derived from those experiences.

**Method:**

Twelve adult players with bereavement experiences (six male, six female, age range: 20-31) played two video games (Bear's Restaurant and Spiritfarer). Their experiences during and after gameplay were tracked via play diaries and in-depth interviews. Data was analyzed using thematic analysis method.

**Results:**

We discovered seven themes on the meanings that players made from their gameplay experience: “Recalling memories”, “Avoiding engagement with the pain”, “Recognizing positive emotions”, “Acknowledging the deceased's perspective”, “Reviewing the meaning of loss”, “Planning a better future”, and “Fulfilling a wish”. Our findings indicate that bereaved players recalled and related their autobiographical memories to their in-game experiences. Furthermore, they derived new meanings on both the loss and their post-loss life after playing video games.

**Discussion:**

We discuss how video games can provide a unique meaning-making experience to bereaved players by affording them an agency to actively reconstruct their narrative of loss and facilitating the sharing of grief.

## 1. Introduction

Life can be challenging—especially when the loved ones who made life worth living pass away. Although bereavement is an inevitable and universal event, losing loved ones to death remains one of the most stressful life events that can detriment both physical and mental health (Holmes and Rahe, 1967; Stroebe et al., 2007), cause intense psychological pain, and bring emotional turbulence (Zisook and Shear, 2009). Adapting to loss involves a complex task where the bereaved must make meaning of their losses and simultaneously manage the cognitive and emotional distress caused by the event (Stroebe et al., 2017). Our previous study Eum et al. (2021) found that grieving players derived new meanings of their loss while playing a video game *Spiritfarer*. Our findings also posed a question on the mechanism of meaning-making experience that playing video games can afford to the grieving players. How and why can playing video games be helpful for adapting to the death of loved ones?

### 1.1. The role of meaning-making in adaptation to loss

After losing their loved ones, bereaved individuals adapt to the loss in different trajectories. Most individuals successfully integrate the loss within 6–18 months and some even experience posttraumatic growth (Bonanno et al., 2002; Dutton and Zisook, 2005; Calhoun et al., 2010; Galatzer-Levy and Bonanno, 2012). However, about 10% of the population experience prolonged symptoms of grief that can harm physical and mental health (Prigerson et al., 2009; Shear et al., 2011; Szuhany et al., 2021).

Constructionist approach to bereavement argues that individual differences in the adaptation to loss depend on how successful the individuals are in their search for meaning in the loss. In this approach, the loss is understood as an event that challenges bereaved individuals' core beliefs about the world and threatens the coherency in their self-narrative. The meaning can be reconstructed through an iterative process of avoiding and confronting one's emotions, memories, and beliefs related to the loss; integrating the loss into his or her personal narrative through this work is deemed essential for adaptation to loss (Neimeyer, 2001). Through this process, individuals can make sense of the loss, find benefits in it, and even experience a change in self which is associated with adaptation to loss (Gillies and Neimeyer, 2006; Holland et al., 2006).

Recent research on fostering meaning-making in the context of loss has expanded to cover a wide range of topics. One significant area of focus is the sharing of grief, which can be an effective approach to reconstructing meaning and reducing grief levels. In fact, correctly assessing emotional reactions to autobiographical memories of loss is closely linked to grief reactions (Lobb et al., 2010; Mason et al., 2020), and remembering specific episodes and emotions in a self-focused and positive manner is associated with lower levels of grief (Eisma et al., 2015; Wolf and Pociunaite, 2018; Smith and Ehlers, 2020; Wolf et al., 2021). Constructivist grief therapies encourage bereaved individuals to confront their emotions and memories of the loss by retelling their bereavement experience, focusing on the metaphorical language, and engaging in evocative visualization of their emotions (Neimeyer et al., 2010; Neimeyer, 2019). Recent studies also highlight the importance of sharing grief within a community of close individuals who have experienced the loss together, such as family, for the co-construction of meaning during the adaptation process (i.e., Barboza et al., 2022). However, the cultural and relational context can often make disclosing grief challenging (Hooghe et al., 2018; Li et al., 2023). Interactive technologies like chatbots have shown promise as a novel way to assist the integration of the loss by providing a virtual presence for sharing grief experiences, resolving unfinished issues, and supporting identity reconstruction (Xygkou et al., 2023).

### 1.2. Players' experience of meaning-making of loss through video game play

Recent game studies focused on the potential of video games to provide meaningful experiences for players. In particular, players seem to derive meanings from video games by either players attributing personalized significance to in-game experiences or establishing direct links between in-game experiences and real-life challenges (Daneels et al., 2021). Empirical studies showed that video games can generate diverse meaningful experiences such as complex emotional and reflective experiences through relating in-game experiences and out-of-game experiences (Bopp et al., 2016; Mekler et al., 2018). Also, video games can provide a distant space where players can actively explore their emotions, reflect on their past and present life experiences that resonate with their in-game experiences, and find new meaning and values (Bopp et al., 2021; Spors and Kaufman, 2021).

The potential of using meaningful gaming experiences to facilitate bereaved players' meaning-making of loss has received little research attention. Empirical studies suggest that playing video games provided a lifeline for bereaved players overwhelmed by grief (Iacovides and Mekler, 2019; Bopp et al., 2021), and designing games has been explored as a medium for bereaved individuals to construct their own self-narrative on loss and share their grief experience (Harrer, 2018). However, only a few studies have explored how grieving players made new meanings of their loss through playing video games. They are mostly case studies on a single game, such as *Mandagon* and *Spiritfarer*, and their findings suggest that playing these games can help players reflect on their loss by making players engage with a gameplay experience similar to actual grieving experience (McGuire, 2020; Eum et al., 2021). Specifically, in our previous study, we discovered that playing the game *Spiritfarer* helped bereaved players reflect on the memories related to their lost loved ones and reappraise their loss in a more positive manner by engaging them with the simulated bereavement experience with the in-game characters (Eum et al., 2021). However, the specific trajectories for the meaning-making of loss through playing these games were not examined in detail, and it is unclear whether similar experiences can be obtained from other games with similar themes. Therefore, further research is needed to explore the mechanisms and potential benefits of gameplay in the context of meaning-making of loss.

### 1.3. Research question

Based on the findings from our previous study, we assumed that the bereaved players will encounter in-game scenes which would elicit autobiographical memories related to the loss (i.e., memories about the deceased or coping with the loss). We also presumed that by linking their in-game experiences to their personal memories, players will be able to engage in the meaning-making of their loss. In this study, we aimed to explore whether the above assumptions would hold true for the players with loss experiences who play two different commercial video games about death and bereavement. We set three research questions according to the steps players undergo to reflect on the in-game experiences and relate the out-of-game experiences to them:

1. What autobiographical memories did players recall as they played *Bear's Restaurant* and *Spiritfarer*?
2. How did players reflect on the autobiographical memories they recalled in two games?
3. What meanings did players make of their gameplay experience after playing two games?

The rest of the paper is as follows. We explain participant information, study procedure, characteristics of chosen games, and ethical considerations in the Methods section. We then present the seven themes that arose from the thematic analysis of the diaries and interview transcripts in the result section. The discussion section provides the interpretation of the results in relation to prior literature and presents our contributions to related fields of research.

## 2. Methods

### 2.1. Participants and recruitment

We recruited 12 participants (six male, six female, age range 20–35 years, mean age = 25.42) for this study. All participants were undergraduates or graduate students from the nearby campus area of the authors affiliation. They were recruited via flyers posted on school community websites. The recruitment criterion was that none of them played *Spiritfarer* or *Bear's Restaurant* before, and they have experienced the death of at least one family member, relative, friend, or a pet. In addition, we ensured to recruit participants who were not receiving medical treatment for depression, anxiety disorder, or prolonged grief disorder. Participants who scored above the clinical cutoff score on the depression (PHQ-9; Kroenke et al., 2001) and the anxiety self-report measures (GAD-7; Spitzer et al., 2006) in the recruitment survey were rejected. We also implemented the Inventory of Complicated Grief (ICG), a self-report scale for assessing complicated grief (Prigerson et al., 1995). Participants whose bereavement happened more than 12 months ago and scored over the cutoff score on the ICG were also informed of their results and rejected. This was to protect them from the potential mental health risks of this study. After participation, participants were compensated with 100,000 KRW.

Participants' bereavement contexts were collected using the 13 items in the Texas Revised Inventory of Grief that measure present grief (TRIG-Present; Faschingbauer et al., 1981, cited from An, 2006; Holm et al., 2018). Participants varied in their age, nationality, and bereavement context such as the number of losses they experienced in their life, their relationship with the deceased, time since loss, and perceived psychological closeness to the deceased differed across participants. We intentionally kept the diversity in nationality and bereavement context for two reasons. First, diversity was deemed necessary to capture recurring patterns in the different trajectories of in-game and post-game experiences. Second, the previous study (Eum et al., 2021) showed that the players with varying bereavement experiences reported different responses during gameplay. We also collected bereavement contexts such as the relationship to the deceased, the cause of death, time since loss, and the perceived psychological distance to the deceased which were known to influence adaptation to bereavement (Bonanno and Kaltman, 2001; Zisook and Shear, 2009; Lobb et al., 2010). Table 1 illustrates participants' demographics and bereavement contexts.

**Table 1:** Participant demographics and bereavement contexts.

**Name**	**Age range**	**Nationality**	**Time since the most recent loss**	**Perceived psychological closeness to the deceased**	**Relationship to the deceased**	**Nature of death (cause of death)**
P1	24–27	South Korea	5–10 years ago	About as close as most of my relationships with other people	Uncle Pet	Sudden (illness)
P2	24–27	South Korea	5–10 years ago	Closer than most relationships I've had with other people	Grandparent Friend	Slow (illness), Sudden (accident)
P3	20–23	South Korea	5–10 years ago	Closer than most relationships
I've had with other people	Grandparent Pet	Unexpected (illness)
P4	28–31	South Korea	6–9 months ago	Closer than most relationships I've had with other people	Grandparent	Unexpected (accident)
P5	28–31	South Korea	5–10 years ago	Closer than most relationships I've had with other people	Grandparent Friend	Unexpected, sudden (illness)
P6	20–23	South Korea	2–5 years ago	About as close as most of my relationships with other people	Grandparent Uncle	Slow (illness) Sudden (accident)
P7	24–27	Indonesia	6–9 months ago	Closer than most relationships I've had with other people	Grandparent Aunt	Unexpected, sudden (illness)
P8	20–23	India	3–6 months ago	Closer than most relationships I've had with other people	Friend	Unexpected, sudden (accident)
P9	20–23	US/German	10–20 years ago	Closer than any relationships I've had with other people	Parent	Unexpected, slow (illness)
P10	24–27	Ethiopia	3–6 months ago	Closer than any relationships I've had with other people	Uncle Aunt	Unexpected, sudden (accident) Unexpected, sudden (illness)
P11	28–31	Mexico	1–2 years ago	Closer than most relationships I've had with other people	Grandparent Pet	Slow (illness)
P12	24–27	Bolivia	9–12 months ago	Closer than most relationships I've had with other people	Grandparent	Slow (illness)

### 2.2. Game choice

For this study, we chose two games: *Bear's Restaurant* (Odencat, 2021; BR) and *Spiritfarer* (Thunder Lotus, 2020; SP). In both games, players have to progress the story by completing quests given by spirits in the afterlife. The two games support multiple platforms (PC, console, and mobile) and *Spiritfarer* affords local co-op play other than the default single-player mode. However, we asked participants to play the PC version in this study in order to reduce differences in players gameplay experiences that may occur from using different hardware or play modes.

We selected two games based on their ability to evoke autobiographical memories related to the loss. The games' themes and character stories are expected to provide numerous potential links to players' memories related to the loss, as both games' narratives explicitly focus on death and bereavement with diverse in-game characters that players can relate to people who have passed away. Our prior research and developers' commentaries support the idea that playing these games can help players recall memories of loved ones (Daigo, 2021; Escapist, 2021; Eum et al., 2021). Moreover, the main tasks in both games involve actions similar to the actual bereavement process, which can encourage players to relate their in-game experiences to their own memories. Players form relationships with the game characters in both games by completing quests that reveal their past, cause of death, and final wishes. However, once the player discovers the character's full story, they are required to send them away, leaving only a memento (i.e., the character's home in SP or memory shards in BR). Players are unable to interact with the character again, which mirrors the disconnection from the deceased experienced by bereaved individuals.

The selection of the two games was also based on their ability to provide players with the necessary time and space for reflection. Video games often present a challenging environment where players have limited time to analyze in-game experiences in relation to their real-life experiences (Juul, 2019; Atkinson and Parsayi, 2021). However, the two games we chose offer a different type of gameplay with relatively low demands. There are no time limits, win-or-lose conditions, or penalties in the games, and most in-game rewards are not tied to in-game progress. These conditions satisfy the requirements for the reflective mode of playing mentioned in Possler and Klimmt (2023) where players can interpret in-game experiences as symbolic representations of their memories of loss and reflect on their meanings. This reflective mode of play is particularly conducive to the interpretation and reappraisal of personal experiences, as it allows players to take their time and make connections between in-game events and their own lives.

**Figure 1:**
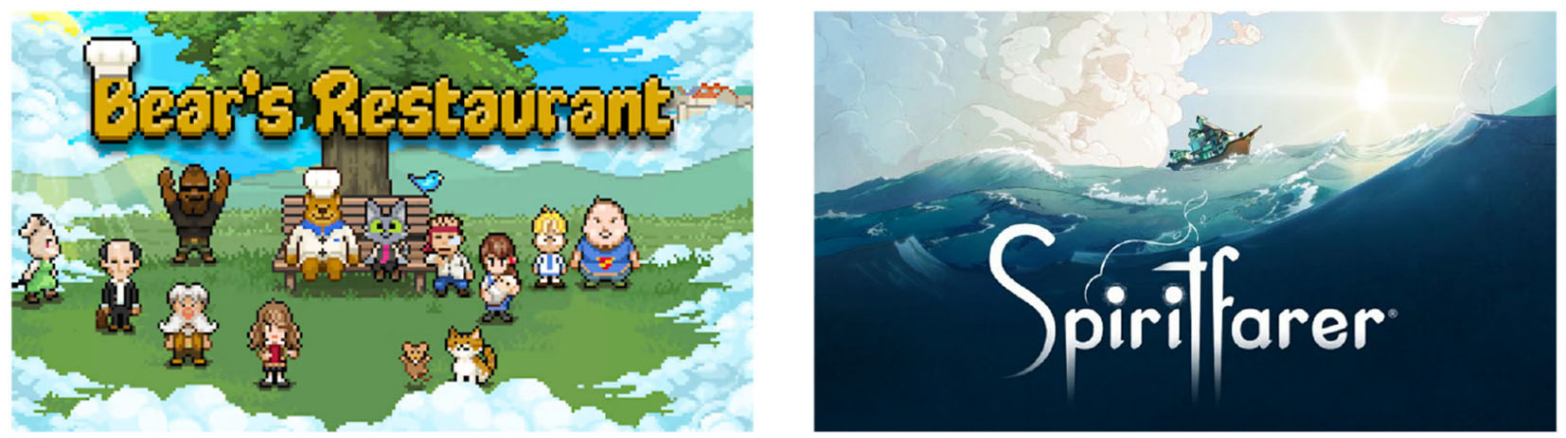
Title images of the two games, *Bear's Restaurant* by Odencat and *Spiritfarer* by Thunder Lotus. Images reproduced with permission of Odencat **(Left)** and Thunder Lotus **(Right)**.

Lastly, we selected the two games based on the assumption that, in reference to the notion of game affordance (Eden et al., 2018), the structural differences in the two games' environments (actions, tasks, and reward structure) would shape players' reflection experience in a different manner. BR affords players more time to contemplate the visual world and the narrative of the game, as most of the quests involve simple clicks to progress the story and the tasks given to players are less in quantity and complexity. The game also provides opportunities to more actively engage with the memories of in-game characters by letting players “dive” into characters' memories and interact with characters to complete the quest or see cutscenes of how they died. Since players can more directly engage with events related to characters' life and death in BR, we expected that players would more likely associate in-game events with real-life events and evoke stronger emotional engagement with the recalled memories. Meanwhile, SP provides players with more complex mechanics and a larger number of quests, requiring players to collect and craft diverse resources in between the journey of discovering the stories of in-game characters. Also, the backstories of in-game characters are implied in the dialogues. Therefore, players are presumed to report smaller frequencies of associating in-game events with their life events, resulting in less recalled memories and weaker emotional engagement with them. Figure 1 shows the title images of the two games; The game studios of the two games, Odencat and Thunder Lotus, have granted the permission to use the images. Table 2 illustrates the structural aspects of the two games.

**Table 2:** Comparison of *Bears' Restaurant* and *Spiritfarer*.

	***Bears' Restaurant***	***Spiritfarer***
Developer (country)	Odencat (Japan)	Thunder Lotus (Canada)
Genre	Indie, adventure, and interactive fiction	Indie, adventure, management, platformer, and farming simulation
Length	3–4 h	40–60 h
Main task	Discover and serve the meal that spirit wants before they leave	Fulfill spirits wishes and take them to Everdoor when they are ready
Actions and quests	Walk around Talk and hand food to characters Dive into characters memories via memory shards	Find new regions Care for spirits onboard (cook and give food, hug, fulfill their request) Gather resources in different regions (mine, fish, and collect) Craft materials out of resources Manage plants and animals in the farm Upgrade ships at the workshop
Narrative	Cat (player character; PC) works in a restaurant in the afterlife that is run by Bear. In hopes of finding her lost memories, she serves spirits the last meal they want to have before they go to heaven or hell. In the process, she finds out about spirits life before they died as well as her lost memories.	Stella (player character; PC) takes a new job as a Spiritfarer in the afterlife where she should find spirits who are not ready to leave the afterlife and take them to Everdoor when they are ready. In the process, she learns spirits memories of their life before death and discovers who she was before she arrived at the afterlife.
Reward structure	Completing quests lets the PC progress the main story and unlock Steam achievements.	PC can gather “glims” from completing quests and “Spirit Flowers” from spirits that left to Everdoor, and use them to upgrade ships in order to discover new regions and stories. Completing quests also unlock Steam achievements.

### 2.3. Procedure

#### 2.3.1. Gameplay week: collecting players' diaries

After the researcher obtained written consent from all participants, players were asked to purchase the two games and start playing for 7 days. We did not limit the date and time they played in a given week in order to capture players' thoughts and emotions in the most natural setting possible. Considering the different playtime of the two games, we set the minimum condition that should be satisfied to participate in the interview: seeing the ending of BR, and sending at least two spirits to Everdoor in SP. The estimated playtime for satisfying the minimum condition was 10–12 hours.

During the given week, participants had to write diaries every day they played two games. In the diary, they attached screenshots of the game scenes that were particularly meaningful to them in relation to their life experience, and answered two open-ended questions. The questions asked why this scene was meaningful to them in relation to their life experience, and what they thought or felt when they encountered the scene. In order to encourage players to freely write their thoughts and feelings, we did not put any limits on the length and number of diaries. The diaries were written online using individual Google Docs documents, which were shared only between the participant and the researcher in order to keep the content private. The play diary examples given to the participants are attached as Supplementary material.

#### 2.3.2. Post-gameplay interview

Participants who satisfied the minimum condition in a given week were interviewed individually. The in-depth interviews were conducted for approximately 1.5 hours via Zoom considering the COVID-19 restrictions. We provided the interview questions to participants prior to the interview. However, since the interviews were semi-structured, some questions were added or elaborated during the interview depending on the participants' answer.

The interview questions asked participants' gameplay history and motivations, their experiences with the two games, what they perceived differently in the two games' environments and their behaviors, and the change in their thoughts or feelings about their bereavement experience. During the interview, participants read their play diaries with the interviewer and discussed if there are memorable scenes that they want to add. Afterward, they chose the scene that was most meaningful to them and explained the reason for their choice. The interviewer also asked questions that asked deeper about the feelings and thoughts described in the play diary when deemed necessary. This was to compensate for the different depth of play diaries across participants and to collect additional data that can help the researchers better understand what they wrote in the diary. The full interview question list can be found in the Supplementary material.

#### 2.3.3. Data analysis

The diaries and interviews were analyzed using Nvivo software. We deemed thematic analysis to be appropriate due to its capability to capture recurring patterns across players' subjective answers (Braun and Clarke, 2006). We used the inductive thematic analysis method in reference to Terry, Hayfield, Braun, and Clarke (Terry et al., 2017).

The analysis was conducted in the following fashion. First, all interviews were transcripted and play diaries were collected in a single Excel file with each sheet containing all diary entries of an individual player. The observational notes taken during the interviews were also reviewed in order to ensure no information was omitted during the transcription. The first author, who is a Native Korean speaker and a proficient English speaker, inductively coded the data using NVivo software. No new codes were generated after the coding of the 11th participant's interview, which was deemed as a sign that the saturation point was reached in reference to Saunders et al. (2018). Afterwards, the first author constructed candidate themes from the codes. To guarantee the credibility of the analysis, we employed the researcher triangulation approach (Nowell et al., 2017) when we reviewed the candidate themes. The candidate themes were subject to multiple triangulation sessions where they were reviewed by all authors and a fellow game researcher with expertise in Game Studies. Researchers reviewed the candidate themes and assessed whether they properly reflected players' experiences. Candidate themes underwent iterative reconstructions during the triangulation process. For instance, the initial candidate themes were differentiated according to the types of recalled memories (emotion-related memory and event-related memory), but they were reconstructed following the feedback that it is hard to distinguish between two memory types in participants' accounts. In the end, seven themes, 30 subthemes, and 135 codes were generated. The finalized themes and their descriptions can be found in Table 3.

**Table 3:** Summary of the themes.

**Theme**	**Category description**	**Conceptual link to the meaning-making of loss**	**Example quotes**
Recalling memories	Participants recalled long-forgotten events, thoughts, and emotions related to the loss	Raising awareness on the presence of unresolved thought or emotion	(The game scene) reminded me of my friend's funeral...(P2)
Avoiding engagement with the pain	Participants sought to distance themselves from the memories after confronting the pain of loss	Part of the iterative process of avoiding and confronting emotions and thoughts on the loss	I don't want to even watch it or see (the game scene) again... It's quite scary. (P10)
Recognizing positive emotions	Participants became aware of the positive emotions in both pre- and post-loss lives	Part of the iterative process of avoiding and confronting emotions and thoughts on the loss	I remembered a lot of my grandmother. It's nice to remember the respect I have for her. (P11)
Acknowledging deceased's perspective	Participants witnessed the loss narrated from the deceased's perspective and reevaluated their loss	Incorporating a new perspective into one's own personal narrative of loss	I came to think about what it would have been from their point of view... (P3)
Reviewing the meaning of loss	Participants confronted the bitterness elicited by recalled memories and reassessed the loss with their current belief on life and death	Integrating the loss and reconstructing a coherent self-narrative	After being sad and nostalgic and bitter... it also makes me think, so what if it didn't happen? (...) a sense of telling myself everything has a reason. (P9)
Planning a better future	Participants shifted their focus from their regrets to cherishing the present and discovering future priorities in life	Restructuring priorities in life to create more coherent self-narrative	I will try to correct my mistakes and give my family appropriate time... (P8)
Fulfilling a wish	Participants resolved the unfinished business with the deceased and experienced peace	Continuing bonds as a resource to continue the meaning-making process	It's something I wish I could hear from my (late) grandma. It made me thankful and relieved. (P7)

### 2.4. Ethical considerations

The research process was approved by the institutional review board (IRB) of the authors affiliation. The study design included measures to protect participants from further distress. The registration survey included self-report measures for depression, anxiety disorder, and complicated grief. Applicants whose score for each self-report measure was above the clinical cutoff score were informed of the result and were rejected from participation in order to screen the applicants with high risk for mental health issues that may arise from participating in this study. In addition, the survey addressed the potential risks of participating in this study and the trigger elements in both games, such as the portrayal of suicide, murder, and illness, and asked if they agreed to proceed despite the warnings and agreed to share their experiences with the researcher. Participants were informed they can stop participating anytime if they experience too much emotional distress in the process. The interview process followed the guide on the interviews on sensitive topics, which contains instructions such as giving open-ended questions and creating a comfortable environment by allowing participants to choose the location and ensuring privacy (Elmir et al., 2011). In addition, in consideration of the potential emotional risk that researchers who study sensitive topics may face (Moncur, 2013; Waycott et al., 2015), several support mechanisms were installed in the study procedure. The corresponding author regularly met with the first author and monitored the study design and interview process. The first author received regular counseling during the study in order to monitor potential emotional difficulties that may arise from the emotional engagement with the topic.

## 3. Results

We found seven different themes from the participants experiences during and after playing two games. Each theme illustrates the types of recalled autobiographical memories, participants' emotional responses to recalled memories, the meanings participants made of in-game scenes, and the meanings participants made of the entire gameplay after they finished playing. While the first three themes showed players' reaction to confronting the memories related to loss, the latter ones showed players deriving new meanings of the loss. Korean participants quotes were translated into English. If participants mentioned an episode from a specific game, we wrote the game title in the parenthesis after the quote. If the participants mentioned their impressions on the two games in general, we did not write the game title to prevent confusion and wrote participant's name only.

This section also presents the screenshots of the in-game scenes that players mentioned in the quotes. We mostly used screenshots taken by the participants in the two games, but in case players forgot to or intentionally did not take in-game pictures, we substituted them with the screenshots taken by other players or the first author. The source of each image is mentioned in the caption. The developers of *Bear's Restaurant*, Odencat, and *Spiritfarer*, Thunder Lotus, have granted the permission to use the screenshots from their respective games.

Table 4 presents the frequency of the codes and their quantitative percentage in each theme, rounded to the nearest whole number. Themes with more subthemes may have a higher percentage even if they were reported by fewer participants, as the frequency is calculated as the sum of subtheme frequencies. To counter this bias, we also noted the number of participants represented in each theme.

**Table 4:** Quantitative percentages of the themes.

**Themes and subthemes**		**Frequency**	**Percentage**	**Participants**
**Recalling memories**		**14**	**10%**	**4**
	Recalled related memories but no further meaning-making	14		
**Avoiding engagement with the pain**		**7**	**5%**	**2**
	Recalled the unexpectedness of the loss	3		
	Surprise	1		
	Shock	3		
**Remembering positive emotions**		**24**	**18%**	**6**
	Recalled fond memories with the deceased	4		
	Recalled fond memories with the living close ones	10		
	Nostalgia	5		
	Gratitude	5		
**Acknowledging the deceaseds perspective**		**28**	**21%**	**4**
	Recalled memories with the deceased	12		
	Regret	7		
	Relief	2		
	Imagined how the deceased felt	4		
	Found benefit in newly discovering the deceased	3		
**Reviewing the meaning of loss**		**35**	**24%**	**5**
	Recalled memories of coping with the loss	13		
	Bitter and longing	4		
	Assessment of belief	9		
	Reappraisal of the past	7		
	Found benefit in sharing my interpretation with others	2		
**Planning a better future**		**22**	**16%**	**5**
	Recalled memories of not spending time with the deceased	3		
	Recalled memories of not spending time with living close ones	5		
	Regret	4		
	Not repeat the mistake and cherish the present	4		
	Positive outlook on the present and the future	6		
**Fulfilling a wish**		**7**	**5%**	**2**
	Recalled own wishes involving the deceased	3		
	Relief	3		
	Gratitude	1		

### 3.1. Recalling memories

In the game, participants discovered various objects or scenes that they could associate with the people in their lives. These objects or scenes elicited the memories of the deceased they had long forgotten (i.e., distant friend). Participants recalled that these experiences brought these losses to their awareness. For instance, P2 remembered a childhood friend who died of an accident when he saw characters playing with a soccer ball in BR, a loss he had long been unaware of:

When I saw that ball, I remembered my friend in elementary school. He chased the ball outside and never made it back. Usually, I dont recall him when I see a ball, but maybe the topic of this game (death) brought the memory back. (P2, BR)

P2 also identified a similarity between the scene from SP and a specific episode related to his loss. He described how the reactions of other characters to the departure of the character who was about to pass away greatly resembled his recollection of his peers' reactions at the funeral:

(The scene) reminded me of my friends funeral. Because he liked the school very much, the bus carrying his coffin drove around the school one time after his funeral and all students paid respect to him. I remembered that moment because we were standing just like that. No change in expressions, just standing (...) I knew what he wanted at his last moments so I think I remembered the scene at the funeral more than who he was. (P2, SP)

Meanwhile, some participants mentioned that game scenes that elicited emotions similar to what they felt at the time of bereavement triggered the memories. P3 described that scenes from both games reminded her of how she felt when she heard about her grandfather's death:

The two characters had a conversation after the reunion and then the game ended like this (a black and white drawing of an empty chair below a tree). I felt like I was really saying goodbye. I thought it was similar to how I felt when I realized the disconnection. (P3, BR)

I was harvesting and planting something on the boat and building a new building. When you're ready to send your soul to Everdoor, the graphics turn into red rivers and trees (...) Somehow, I satisfied the condition to send Alice to Everdoor, so as soon as I woke up, the background color changed. I was really surprised because I didn't do anything. What did I do? (...) I wasn't ready and I was surprised that such events happened again in a row. Although she was a character, I wanted to tell her not to go. (...) I think it was more reminiscent of my experience of being confused when I got a call. (P3, SP)

While both participants recalled the memories of the related episodes and emotional reactions to loss, whether they engaged in further meaning-making of them differed. P2 explained that he did not reflect deeper about his friend's death because he did not have other significant memories associated with the deceased that he could relate to the game scenes. Meanwhile, P3 derived further meanings from his grandfather's death as she repeatedly encountered game scenes that elicited memories of him, as depicted in the theme “Acknowledging the deceased's perspective.”

### 3.2. Avoiding engagement with the pain

**Figure 2:**
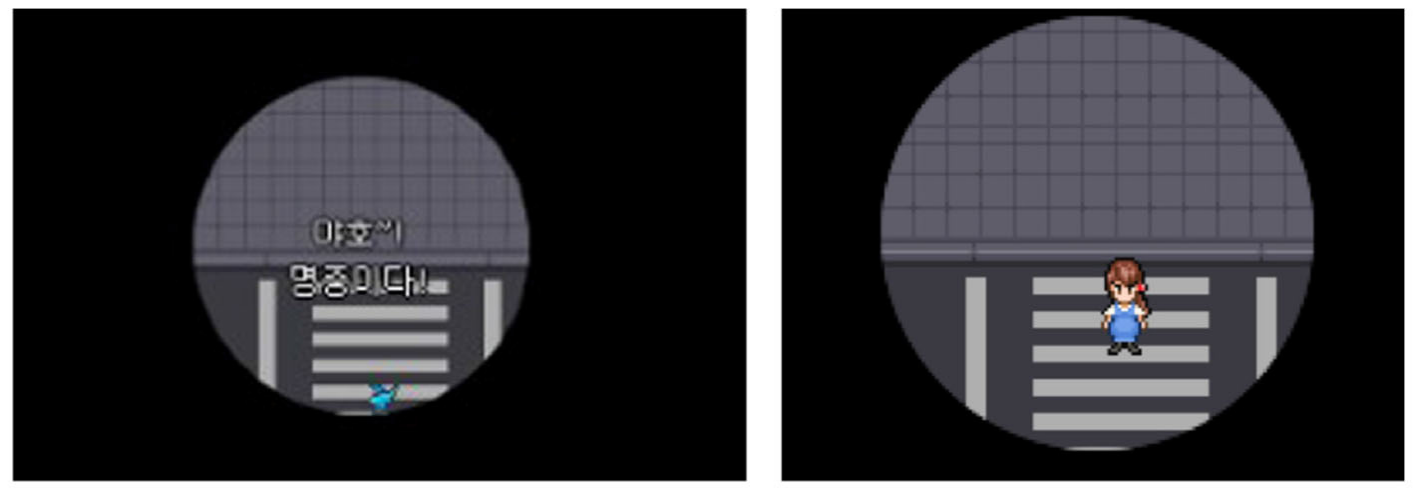
The Bear's Restaurant screenshots taken by participants in the theme “Avoiding engagement with the pain” [**(left)** P1, **(right)** P10]. The screenshot for P10 was substituted by the screenshot of the same scene taken by another participant because she did not capture the scene at the time of gameplay. Reproduced with permission of Odencat.

Recalling memories related to the loss also confronted participants with the pain of losing their loved ones. Some participants reacted with shock and sought to distance themselves from those memories rather than dwelling on them. Two participants reported that they could relate the game scenes to their own experiences because they reminded them of the initial shock they felt upon their loss. The scenes were mostly death scenes that bore similarities to their loved ones cause of death. Those participants shared two bereavement contexts: they were emotionally close to the deceased, and their death was sudden and unexpected. The unexpected bereavement left them with great shock and regret that lasted until the time of the study. The scenes that participants mentioned in the quotes can be found in Figure 2.

When I heard the gunshot I had goosebumps (...) I remembered that I raised a chick when I was young and that I felt very scared and guilty when it died, just like when I saw the bird die in the game. (P1, BR)

I lost my uncle because of a car accident (tears). I don't want to even watch it or see it again. (...) I know how it feels for the parents or for any family (whose member) died in a car accident. It's quite scary. (P10, BR)

The intensity of participants' reactions varied according to the time passed since bereavement. For instance, P1, who lost her pet more than 10 years ago, was surprised but it did not last long. Meanwhile, P10, who lost her relative 2 years ago, was horrified and refused to record the scene in her diary. Despite their differences, both reported avoiding further reflection on their painful memories.

### 3.3. Recognizing positive emotions

Interestingly, the games did not only bring sad memories but also made participants aware of the positive memories in their pre- and post-loss lives. Participants remembered events that accompanied positive affect, such as the good times they spent with the deceased or the close ones who were still alive. Often, the characters lines that described their past life in a positive manner or the scenes that visually matched participants' memories became the cue for retrieval. In the recollection process, the memories were reconstructed focusing on how participants themselves helped the deceased or how joyful the moments they spent together were. Participants reported that they felt nostalgic after recalling memories with the deceased, but no further cognitive processes followed. For instance, P11 happily recalled his final days with his late grandmother when he took care of the character in SP:

In that particular scene, I remembered a lot of my grandmother. It's nice to remember, like the respect I have for her (...) So every step with the hedgehog was like remembering the steps I went with her. It's pretty nice (...) I didn't consider that I was taking care of her. I just put myself to work to make her laugh every day or make her smile. (P11, SP)

Meanwhile, participants also recollected memories of times when living close ones such as family or friends supported them in the hard times. The depictions of characters helping one another or being surrounded by loved ones reminded participants that there were people who cared for them and supported them through dark times. Recognizing the presence of loved ones in their life led to realization that they were not alone and their life is full of positive memories. Participants such as P6 and P8 reported feeling gratitude and positivity from these recollections:

In the game, one kind act of the cat turned a demon into something good. All he needed was a friend. I feel blessed to have some really nice friends in my life who will always be ready to help me whenever I need them. (P8, BR)

Seeing this scene, I thought about what scenes would unfold in my afterlife. Rather than hateful and unhappy times, I only remembered happy and ordinary moments in my everyday life or the times I spent with my loved ones. So I thought I should make more happy memories in my life than (memories of) hate and anxiety. I felt positive and hopeful that making good memories alone can keep me busy. (P6, SP)

### 3.4. Acknowledging the deceased's perspective

**Figure 3:**
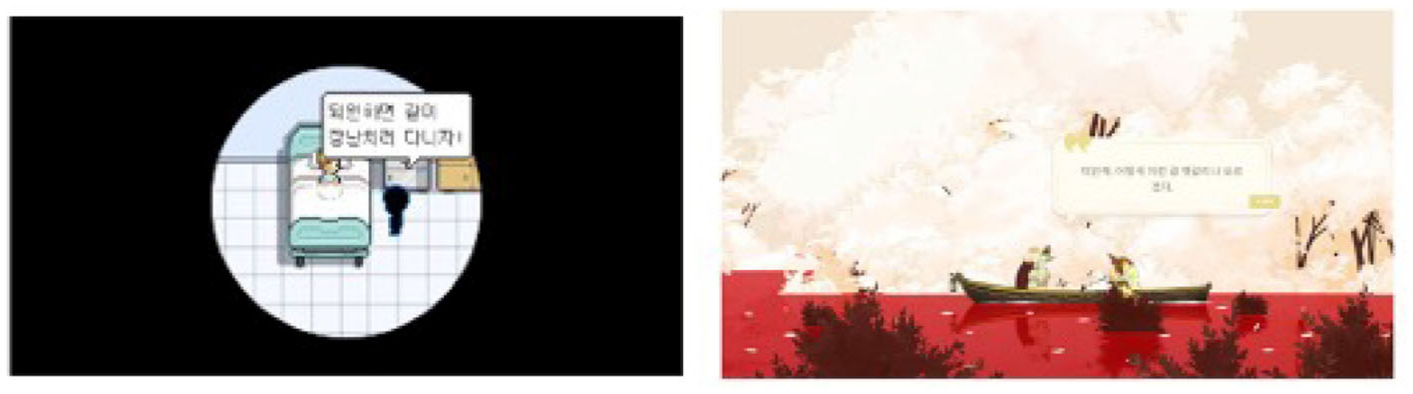
The scenes mentioned in the theme “Acknowledging the deceased's perspective”. **(Left)** P5 from Bear's Restaurant; reproduced with permission of Odencat. **(Right)** P3 from Spiritfarer, reproduced with permission of Thunder Lotus.

Participants also recalled memories with the deceased when they were alive from the specific lines that game characters say or the features of characters stories that overlap with real-life figures. However, while the vantage point of the recalled autobiographical memory was themselves, the game scenes associated with the memories were narrated from the game characters point of view. This created an effect similar to witnessing the same real-life event being narrated from the deceased's perspective. Figure 3 shows the in-game scenes that participants (P5, P3) referred to their quotes.

Participants' reactions differed according to how the event was depicted in the game. For example, both P5 and P7 mentioned that they regretted their interactions with the deceased after encountering a situation in BR that resembled their conversation with the deceased. In the game, they witnessed that characters had regrets before they died, such as not being able to fulfill their last wish or express love to their close ones. Realizing that the past is irreversible, both participants regretted their past behaviors and shifted their focus toward contemplating what they should have said or done to the deceased:

This is the scene where a friend visits the character who dies at the hospital at a young age. I remembered that when I went to see a friend who was years below me and had cancer. He said he wanted to perform on stage, but I discouraged him. I told him to get chemotherapy first, be discharged, and then we can perform together. Thinking back, it could have been his last wish. I was out of line. (P5, BR)

I felt like I was not a good niece, I was not a good granddaughter. Because it seems like I should have done more for them. All this time I only wanted them to understand me. But I never really tried to understand them. Like, what they want, what they wish. I never really talk about those things. Sometimes I also feel like I don't really know them (...) what did I do all this time? (P7, BR)

However, not all participants experienced regret after realizing the perspective of the deceased. Some participants reevaluated memories that were initially associated with negative emotion in a more positive manner after seeing how game characters interpreted their death. This shift mostly occurred in participants who lost their loved ones to illness. For instance, both P5 and P3 lost their loved ones to cancer and related their loss to the negative emotions they felt while witnessing their loved one's health decline. However, as they associated game scenes with their past experiences, they began to interpret their death as salvation from pain, which brought them a sense of relief:

Speaking of this character who died at an early age, my 22-year-old clubmate died of colorectal cancer in a painful way. It occurred to me that he would have been freed from the pain only after he died. (P5, BR)

My grandfather who died of cancer also had some symptoms of dementia. It wasn't that serious, but he began to forget things and his family. I was more taken aback than feeling sad when I saw that. Seeing him confuse my name or mistake me for my younger brother scared me. (...) But even though Alice showed some symptoms (in the game), she looked calm. In that regard, instead of being scared or confused, I thought that it may not have been so painful for my grandfather. It somehow comforted me. (P3, SP)

Not all participants derived different meanings of their loss after gameplay. For instance, P5 commented that he decided to refrain from making judgments on his friend's situation because he deeply regretted how casually he spoke of his friend's last wish. However, other participants who had sufficient time to process grief since the time of bereavement started to realize that their memory of the deceased may not fully capture who the deceased really was. P3 and P7 acknowledged their lack of knowledge about the deceased's true character. They shifted their focus toward the benefits of uncovering and discovering the true nature of the deceased, rather than dwelling solely on the sorrow of their loss. This also involved less rumination and more motivation to share their thoughts and emotions with others who shared memories of the deceased, such as their family:

It made me think more about the ones that passed away. For example, I wonder what kind of things they enjoy, they really do enjoy. Like what kind of things they like and what kind of things they don't like. (...) I want to know them more. (P7)

For too long, I felt that there would be no difference even if I thought about him, so I didn't try to remember him (...) but I came to think about what it would have been like from their point of view. (I thought) maybe it would be okay to think about him a little more. Maybe starting by asking my family what kind of person he was. (P3)

### 3.5. Reviewing the meaning of loss

**Figure 4:**
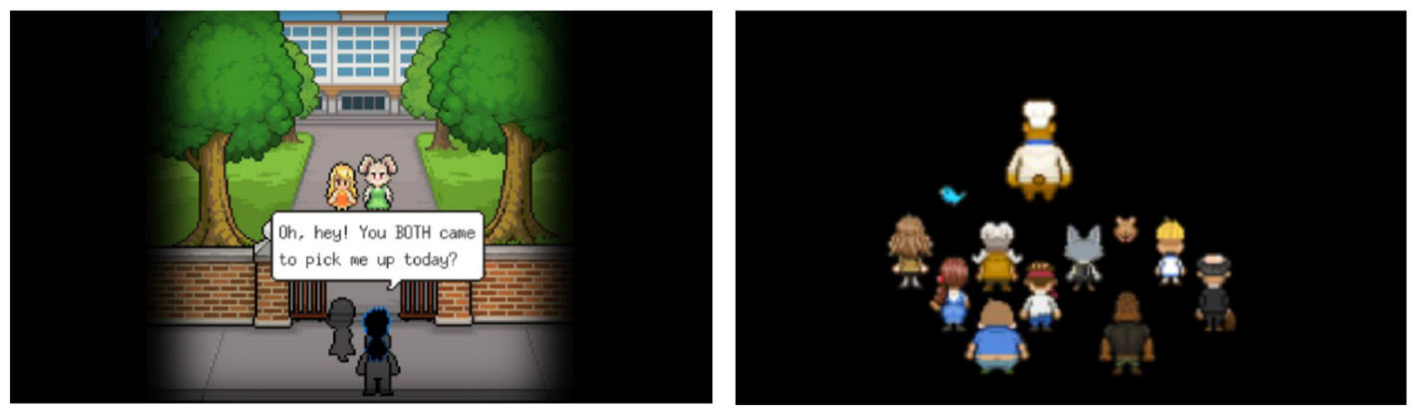
The Bear's Restaurant scenes participants mentioned in the theme “Reviewing the meaning of loss” [**(left)** P9; **(right)** P12]. Reproduced with permission of Odencat.

Participants also recalled events that involved coping with the consequence of the absence of the deceased, such as events during or years after the funeral and what they felt at the time. The memories recreated the bitterness and yearning for the deceased but also acted as a catalyst for further reflections. Reviewing reconstructed memories offered participants an opportunity to reassess past memories with their current interpretation of the meaning of life. Whether participants found a new meaning of loss differed across participants, depending on the discrepancy between the perceived meaning of the game scene and their existing beliefs about life and death. Participants whose interpretation of the games message aligned with their prior beliefs noted that the games served as a time for reassessing their views on life. To quote P4, the games were the review session on how I should live and how I should remember [the deceased]. Figure 4 features the scenes that participants mentioned in their quotes.

Reassessment of ones belief arose during the process of coping with the stress from recalling bitter memories. For instance, P9 recalled the memories that revived strong bitter feelings of grief. However, this was compensated as he reevaluated his past experiences and found benefits in the present:

When I see little kids being picked up by both their parents, or playing with their parents, I always imagined myself being with my deceased dad and how it would have been if he had not died. The memories we would have made together, be it getting picked up from school or just watching a movie together. (P9, BR)

Its like that sad feeling of being like, I wish he wouldn't have to die. I wish I could have experienced life with him. I wish I could have played soccer with him (...) But then after being sad and nostalgic and bitter, it also makes me think, so what if it didn't happen? (...) I made the best of it and I'm happy where I am right now. Maybe all these (new events in life) wouldn't have happened if my dad didn't die. So it's like a sense of telling myself everything has a reason. (P9, BR)

Participants also reassessed their beliefs as they attempted to make meaning of the scenes that contrasted with their prior interpretation of the loss. For instance, P12 discovered a new meaning of her loss when she saw that the spirit characters gathered to help her in BR. While each character had different backstories, they all empathized with the main character's suffering because they shared the common experience of losing something or someone valuable in their life, such as the time with their family or loved ones who passed away. Reflecting on this scene, she realized that, in contrast to her prior belief that no one can understand her pain, grief is a normal reaction that everyone experienced at least once in their lives, including her close ones. The realization broadened her perspective:

When you lose somebody or you feel sad about it, you say nobody can understand what I feel, or this is hard for me and I'm alone. But the reality is, everybody has been through that. Everybody has a kind of experience like that. And it's just nice that you can relate to somebody, to say maybe it's normal to feel like this or to have this process. (...) it liberates you. (P12, BR)

This experience also acted as a catalyst to disclose herself to her close ones. She found benefit in sharing her feelings and thoughts on the loss with others:

As I felt comfortable with these games, like because I was pushed to think about these topics that you usually don't, it just made me think, What if I express myself about these topics more often? Would I feel more relieved or more comforted by the other people who listen to me? (...) Maybe it's a conversation I have to have more often with other people. (P12)

### 3.6. Planning a better future

**Figure 5:**
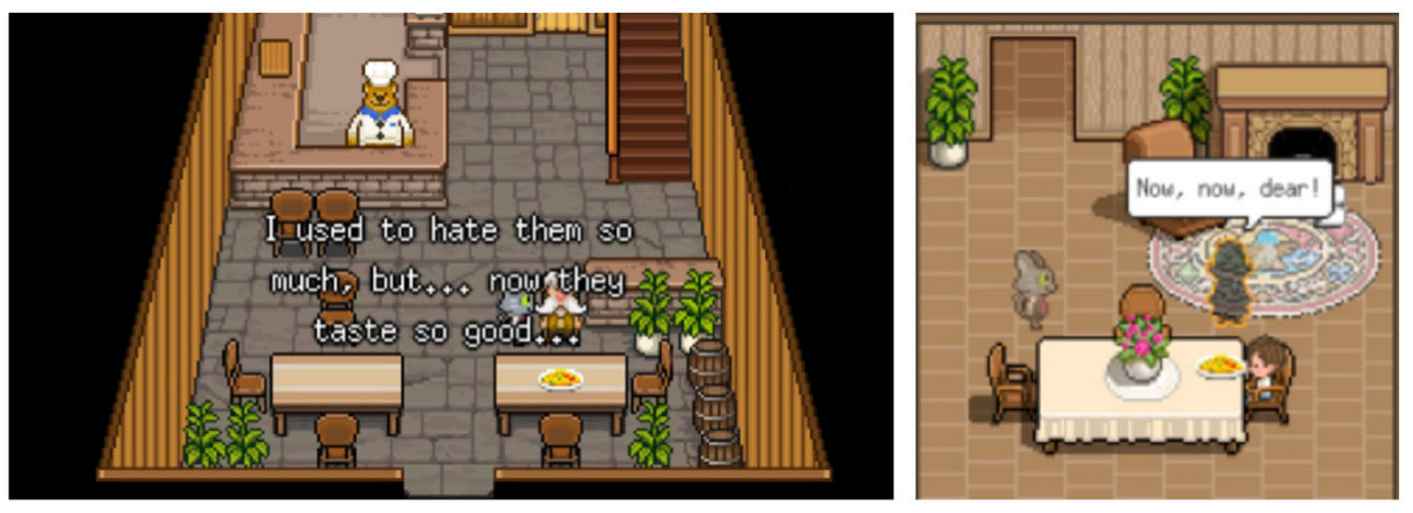
The Bear's Restaurant scenes participants mentioned in the theme “Planning a better future” [**(left)** P8; **(right)** P10]. Reproduced with permission of Odencat.

Participants also reported that confronting their regret motivated them to plan a different future. Game characters' lines or situations elicited their regret for not cherishing the time with their loved ones. In contrast to the previous theme, the loved ones they recalled were not only the deceased but also their living close ones. Participants became aware of the fact that they cannot undo their past mistakes with the deceased, but they realized that they could choose not to repeat the same mistakes with their living close ones. They began to shift their focus from the ones they lost to cherishing the present with their living loved ones so that they can prevent future regrets, as captured by the quotes from P8 and P10. The game scenes mentioned in their quotes can be found in Figure 5.

Just like this man in the scene is regretting not liking food from his mothers' hand, I miss my family sometimes too now that I live in a different country far from them. I regret not spending more time with them, but at least I still have the chance to talk more to them. I will try to correct my mistakes and give my family appropriate time whenever I go back home on vacation. (P8, BR)

This scene reminds me of my mother, she would always cook food for me. And she even knows which one I liked most. After coming to Korea, I regretted not appreciating her help more and I could have been much more affectionate (...) When I meet her in person, I promised to myself that I will be much more grateful and affectionate. (P10, BR)

Being reminded of their past mistakes and planning not to repeat them brought changes in some participants' daily lives after playing two games. More than half of participants spoke of how they began to appreciate small happiness in everyday life, connect more with their close ones, and express their love toward them. For instance, P8 described that playing two games made him more understanding of other people and realize how important family and friends are in his life. He began to make small changes:

After playing these games, I did talk to some of my friends more, like not about the game but like, to connect more with them. (P8)

Even if the game did not greatly change their interpretation of their loss, participants were able to more clearly identify how they felt or thought about the loss. Moreover, it provided participants an opportunity to discover what they would value in their future lives and construct their own beliefs on life and death. To quote P11, playing two games was “not an inflection point (...) but a process” to move on from their loss. P10 suggested that playing two games let her feel more prepared for the future:

I think it changed the fact about the future for me, because what's lost is lost, regret is not that good. (...) Try to remember what you had, happy moments in the past with those people you lost and try to do much better for those you already have. I wouldn't say it made me a different person. But maybe when a situation arises, the way I react to it might be different. It might be to recall those memories, like what I had, what I played, or what I did in these games. (P10)

### 3.7. Fulfilling a wish

Rarely, playing games provided an opportunity to fulfill a wish that involved the loved ones they lost. Their wish was left unresolved because their death was irreversible, but participants were able to fulfill them by interacting with the game characters that they strongly associated with the deceased. This phenomenon was observed in only two participants (P3, P7) because the condition for such a strong association was hard to achieve. First, participants had to encounter characters whose features greatly overlapped with their mental image of the deceased. Second, the interaction that the games afford had to match the interaction they wanted to have with the deceased. In BR, P3 was able to provide a decent meal to a mouse character who she associated with her late pet. In SP, P7 was able to hear the word of acknowledgment of her hard work from the character who shared many features of her late grandmother such as the voice tone, calling her by nickname, and being an old woman.

**Figure 6:**
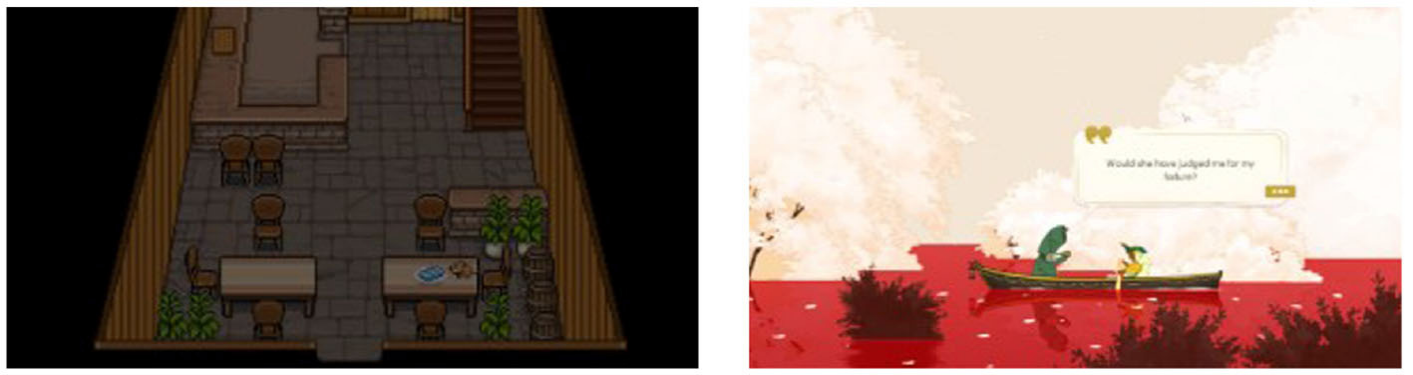
The game scenes participants mentioned in the theme “Fulfilling a wish” [**(left)** P3 from Bear's Restaurant, reproduced with permission of Odencat; **(right)** P7 from Spiritfarer, reproduced with permission of Thunder Lotus]. Since P7 did not take a screenshot of the exact moment, the image was replaced by another participants screenshot at the same scene, but with a different line.

Both interactions elicited relief and a feeling of deep gratitude in players. Playing two games added a new layer of emotion and memory on the meaning of their loss and, more importantly, their life. The specific game scenes that participants mentioned in the quotes can be found in Figure 6.

I raised panda mice as a pet a few years ago. I felt heavy from seeing how characters to whom I served meals died, but suddenly a mushroom-loving mouse appeared, which was cute. I couldnt raise my pet in a proper environment because I was too young and it bothered me since, but giving the mouse its favorite food and sending it off to heaven was comforting. (P3, BR)

Before (going to Everdoor) she said something like “Good job Stella.” (...) It's something that I wish I could hear from my grandma. It made me feel thankful and relieved. Because it means that all the hard work, all the hardships that I've been through, my grandma acknowledged it. So I felt like, “Oh yeah I did it the right thing.” (P7, SP)

## 4. Discussion

This study investigated how bereaved players made new meanings on their loss by deriving meanings from the in-game experiences that elicited personal memories of their loss. We discovered seven themes of the experiences related to the meaning-making of loss by analyzing the interviews with the twelve bereaved players and their diaries: Recalling memories, Avoiding engagement with the pain, Recognizing positive emotions, Acknowledging the deceased's perspective, Reviewing the meaning of loss, Planning a better future, and Fulfilling a wish. We will now discuss the implications of these findings.

### 4.1. Can video games facilitate the meaning-making of loss?

Our results showed that participants encountered various situations in games which elicited memories of loss. Although their reactions to the recalled thoughts and emotions on the loss varied (i.e., “Avoiding engagement with the pain” and “Recognizing positive emotions”), we discovered that participants were able to evaluate and construct their own meaning of loss (i.e., “Reviewing the meaning of loss”). Extending Daneels et al. (2021), participants found the meaning of loss from their in-game experience in two forms. First, as participants relived certain emotions and events in the game, they are able to create direct meanings by connecting them with emotions and thoughts they experienced during the bereavement process. Also, they were able to gain new perspectives on their loved one's death by interpreting in-game situations and attaching new meanings to them.

Interestingly, participants reported deriving generally positive meanings from the in-game experiences (i.e., finding benefit in discovering the deceased, positive outlook on the present and the future). Moreover, participants were able to focus on the meaning-making of their loss without dwelling on their memories too long. This appears to have occurred through multiple mechanisms. First, the games encouraged players to cope with challenges using autobiographical remembering, an act shown to be related to the positive meaning-making of loss (Wolf et al., 2021). Participants experienced emotional challenges in two games as they confronted negative emotions attached to their memories of loss (i.e., P9 from “Reviewing the meaning of loss”), which may have fostered participants to find ways to use recalled memories to cope with current problems and make positive meanings of their experience. Second, the games provided opportunities to recall positive emotions from their memories, which may have compensated for negative emotions from recalling the loss. Particularly, revisiting memories about the living close ones reminded players that they had people who supported them and that they still have time to compensate for their faults, shifting their focus to cherishing the present and finding benefits from the loss. Third, considering that the mood at the time of retrieval could have affected the valence of the retrieved autobiographical memories (Holland and Kensinger, 2010; Konrad et al., 2016), the playful atmosphere that the game medium possesses could have reinforced a positive meaning-making of recalled memories.

### 4.2. The unique meaning-making experience of loss from playing video games

The results of this study suggest that the unique meaning-making experience of video games may be attributed to the aesthetic distance they afford during the process. Aesthetic distance involves adopting the roles of both “cognitive observer (...) (and) affective actor” (Landy, 1994, as cited in Glass, 2006), which is important for the reflection on traumatic events and associated emotions necessary for meaning-making (Glass, 2006). Previous entertainment media studies that dealt with the theme of death and loss suggested that meaningful media can provide an anxiety-buffering function (Rieger and Hofer, 2017) and foster a sense of connectedness (Das and Peters, 2022). Moreover, reflecting on tragedy has been found to be able to promote a deeper understanding of oneself (Khoo, 2016). However, they mostly feature reflective mode of media consumption. In contrast, the two video games seem to have afforded an active and immersive meaning-making experience to participants.

On one hand, the two games provided an environment where participants could make a cognitive evaluation of their loss from a safe distance. Participants were able to contemplate their evaluation of the past events (“Reviewing the meaning of loss”) and restructure their values (“Planning a better future”). The video games' ability to provide a healthy escape where players can distance themselves away from reality (Kosa and Uysal, 2020; Spors and Kaufman, 2021) may have helped participants confront their thoughts and emotions on the loss without being overwhelmed by them or being disconnected from them. It is important to note that selecting games with low behavioral demands would be important to achieve such a reflective mode of playing, as suggested by Possler and Klimmt (2023).

At the same time, the two games also let participants interact with their memories as an active and affective agent. As explained in the Game Choice section, the two games mandated players to engage in a process similar to a bereavement in order to progress. In the lived verisimilitude afforded by two games (Atkinson and Parsayi, 2021), participants were able to relive their past and actively interact with game characters that partly embodied their mental image of the deceased, which provided grounds for constructing new meanings of loss. For instance, participants were able to see the loss from a new perspective (i.e., P3 and P5 from “Acknowledging deceased's perspective”) and resolve unresolved relational issues they had with the deceased (i.e., P3 and P7 from “Fulfilling a wish”). This finding expands on the discussion of how the diverse forms of agency in games shape complex emotional experiences (Cole and Gillies, 2021) and suggests how the agency that games provide can foster bereaved individuals to take an active role in their bereavement journey.

Interestingly, participants appeared to have more easily disclose their emotions and receive social validation of their grief by sharing their in-game experiences to their close ones, which also fostered reconstruction of one's meaning (i.e., P12 from “Reviewing the meaning of loss”). This suggests that sharing loss-related memories and emotions through sharing gameplay experiences can serve as an avenue that bereaved players can use to obtain social support for meaning-making in a less demanding way. This opens venues for interesting future works. For instance, expanding the previous research that showed the bereaved individuals' prevalent use of online support groups to deal with grief (Baglione et al., 2018), future works can explore how video games can be used in support groups to aid the expression of grief or design novel forms of support system for the bereaved individuals.

### 4.3. What other factors may foster further meaning-making of loss through gameplay

It is important to note how the interaction between different game designs and participants' bereavement contexts shaped participants' meaning-making experience. We discovered that the game designs of the two games had a more profound effect on players' experiences than we anticipated. In BR, players directly linked in-game events to their past experiences as we assumed but their emotional responses were not predominantly negative. Rather, the game helped players acknowledge both their positive and negative emotions, leading them to derive positive meanings of appreciating their present lives and futures with their loved ones. On the other hand, we assumed that SP would produce a less frequent association with past memories since it provided more subtle reminders of loss. However, SP also allowed for more vivid reliving of emotional memories and a wide range of emotions from the process of forming relationships with characters and bidding them farewell, which fostered the meaning-making process. Our findings are consistent with a previous study that indicated individual differences in prior life experience affected how players responded to and interpreted their gaming experience (Eum et al., 2021). Specifically, the extent to which they had processed their emotional reaction to loss before gameplay appeared to play a crucial role in their successful reflection through cognitive distancing.

Additionally, players concurred that the task of writing a diary on their screenshots aided them in relating their in-game experiences to memories of actual events. Reviewing and writing on the meaningfulness of the screenshots helped them be more aware of the thoughts and emotions they did not notice during gameplay, which added depth to their meaning-making of past events. We extended the restorative potential of photographing daily moments in grief processing (Jiménez-Alonso and Bresco de Luna, 2022) by demonstrating that taking pictures of virtual experiences could also make participants more aware of the impact of their gaming experience on themselves, in line with the work of Hall et al. (2021). Pairing gameplay with activities that enable players to connect their gaming and real-life experiences may be essential for promoting the meaning-making of the loss in bereaved individuals.

### 4.4. Limitations, future works and contributions

This study comes with limitations. First, we would like to emphasize that this research does not guarantee any clinical implications for treating grief. In particular, how players who were diagnosed with Complicated Grief (CG) might experience target games is beyond our research scope. We cannot exclude the possibility that the games capability to afford vivid, emotional experiences can also provide triggers for negative rumination for players experiencing CG, which needs to be investigated in future works with caution. Furthermore, due to its explorative nature, this study covered a relatively small sample size, and bereavement contexts were not controlled in order to capture the most natural, diverse trajectories of players' experiences. Our findings do not provide the effect size of different patterns nor the correlation of the variables mentioned in each trajectory. Moreover, the saturation was verified by one additional interview, which indicates that a larger sample size may be needed to fully capture bereaved players' experience of two games. Future qualitative studies with a larger sample, possibly with a more controlled bereavement context, may be needed to uncover potential areas that might not have been adequately addressed in this study.

The participants' diaries and interviews may also contain biases worth noting. Similar to studies on positive psychology interventions (Lee et al., 2021), the fact that participants were aware that this study was about adapting to bereavement could have resulted in participants noticing and reporting more changes. Therefore, results should be interpreted with caution. Also, the positive impact of playing games reported in this paper could change when longitudinally monitored. In fact, the long-term experience of playing games may differ from the short-term benefits of playing and return more negative results (Von der Heiden et al., 2019). In line with the increasing importance of longitudinal studies on player experience (Ballou, 2023), investigating the long-term benefits of playing video games for fostering the meaning-making of loss can be an interesting avenue for future research.

Notwithstanding the above limitations, we contribute to a better understanding of how video games can facilitate meaning-making of loss for the players who experienced the death of their loved ones. We found that bereaved players were able to engage with their thoughts and emotions on the loss and make new meanings of them by relating the in-game experiences to their autobiographical memories of loss, expanding the premise of the positive role of playing video games when adapting to stressful life events. Moreover, our findings showcase the unique meaning-making experience that players can experience from playing video games that can elicit memories related to the loss. Participants were able to draw new meanings of loss in the aesthetic distance afforded by the two games; they confronted their thoughts and emotions on the loss from a safe distance, but simultaneously they actively interacted with their reconstructed memories, which provided grounds for constructing new meanings of loss. The impact of bereavement context, game design, and the presence of a journaling activity on the meaning-making experience requires further investigation in future research.

## Data availability statement

The datasets presented in this article are not readily available because participants did not agree to share their private data to the public. Requests to access the datasets should be directed to YYD, yydoh@kaist.ac.kr.

## Ethics statement

The studies involving human participants were reviewed and approved by KAIST Institutional Review Board. The patients/participants provided their written informed consent to participate in this study.

## Author contributions

KE collected, transcribed, and coded all data (in-depth interviews and play diaries) and wrote the manuscript. YYD guided and provided necessary supervision during the research process and reviewed the manuscript. All authors participated in the research design as well as the creation and triangulation of the themes. All authors contributed to the article and approved the submitted version.
