# Supplementary material for: A thematic analysis of bereaved adults' meaning-making experience of loss through playing video games
Source: Front Psychol. 2023 Jul 31;14:1154976. doi: 10.3389/fpsyg.2023.1154976 (PMC10426378; doi:10.3389/fpsyg.2023.1154976)
Supplement: Supplementary file 1 [file Data_Sheet_1.pdf]

Supplementary Material

A Thematic Analysis of Bereaved Adults' Meaning-making Experience of Loss through Playing Video Games

Karam Eum, Young Yim Doh*

*** Correspondence:** Young Yim Doh: yydoh@kaist.ac.kr

1 In-depth Interview Question List (English version)

***To participants: The questions may change depending on what you wrote in the play diary.**

Area 1: Game play context

- How often do you usually play games?
- When do you usually play games?
- What were the most impressive game(s) you played, and why?
- Are there any factors that you value when choosing or evaluating a game? It could be anything, from art, music, story, price, expected playtime, and developers….
- Have you ever played games with heavy topics such as "Bear's Restaurant" or "Spiritfarer" before?

Area 2: Gameplay Experience of "Spiritfarer" (About your play diary)

- How long did you play?
- What was the most impressive / meaningful moment while playing the game? Can you explain in more detail in relation to what you wrote in the play diary?
- Were there any other memorable scenes that you want to add?
- After playing the game, did you think about the deceased more?
- What elements of the game were particularly impressive?

Area 3: Gameplay Experience of “Bear's Restaurant" (About your play diary)

- How long did you play?
- What was the most impressive / meaningful moment while playing the game? Can you explain in more detail in relation to what you wrote in the play diary?
- Were there any other memorable scenes that you want to add?
- After playing the game, did you think about the deceased more?
- What elements of the game were particularly impressive?

Area 4: Comparing the two games.

- Were there any similarities or differences between the experiences you had while playing the two games? What did you feel were similar or different?
- Both games have a core play loop of meeting, caring, and sending spirits away in the game, what did you think or feel about each process?

- Which of the two games did you feel resonated more with your life experience, and why?

Area 5: Attitude towards bereavement experience and death in general

- When you think about death, what thoughts / feelings come to your mind?
- When you look back, what were your thoughts / feelings on the death of your family/relatives/friends/acquaintances/pet before playing the game?
- Were there any changes in your thoughts or feelings about the experience before and after playing the game?
- Were there any changes in your thoughts or feelings about death before and after playing the game?
- Were there any changes in your daily life pattern or perspectives after playing the game? (Thinking more about the deceased, becoming better at understanding the deceased,...) What impact did it have on your days, or yourself?

2 Play diary template and example

2.1 Play diary template

**____ 's Play Diary**	
Game Title: *e.g. Spiritfarer*	Play date & time: *e.g. 2022.03.10 22:00*

Instructions for writing play diary

- The purpose of collecting this diary is to observe how players' thoughts and emotions change as they play the game. Every time you encounter scenes that are meaningful or impressive in relation to your life, take a screenshot of the scene and answer below questions.
- **You can play the game whenever date/ time you are available.** However, you need to satisfy a minimum clear condition for each game in order to proceed to an in-depth interview, which would take about 10 hours. Therefore, I recommend playing 1~2 hours per day regularly.
- **Please write each diary page per each day you play. I also recommend you write a diary within one hour :)** This is because it'll be easy for you to write vividly when you still have fresh memories of the game.
- **There is no page / amount limit for the diary. (no limit for the number of screenshots per each day's diary too)** Please write freely as much as you want.

Play Diary Questions

1. What was the scene that was meaningful / impressive in relation to your life?

2. Why was this scene meaningful / impressive to you in relation to your life experience? What thoughts / emotion did you have when you encountered this scene?

2.2 Play diary example (given to participants for reference)

<table>
<tr><th colspan="2">Example: Julie’s Play diary</th></tr>
<tr><td>Game Title: Spiritfarer</td><td>Play date & time: 2022.03.10 22:00</td></tr>
<tr><td colspan="2">
1. What was the scene that was meaningful / impressive in relation to your life?

Screenshot of the in-game scene

2. Why was this scene meaningful / impressive to you in relation to your life experience? What thoughts / emotion did you have when you encountered this scene?

Seeing how Alice, the grandma hedgehog, recalls her memory with her granddaughter reminded me of the days I spent with my grandmother. Before she passed away, she said she wanted to spend more time with me. When I think back, I used to work or talk about how my work was going even when I was with my family. But I think my grandma wanted to talk more about small things in life, like how my day was going. I felt sorry because I felt I didn’t do what she wanted when she was still alive.
</td></tr>
</table>
